# Clinical trajectories and medication response in *TBC1D24*‐related epilepsies

**DOI:** 10.1111/epi.70013

**Published:** 2025-11-10

**Authors:** Ealing Mondragon, Jan H. Magielski, Bintou Bane, JoeyLynn Nolan, Sarah M. Ruggiero, Dallas Armstrong, Susan Arnold, Deepa Sirsi, Ingo Helbig, Jillian L. McKee

**Affiliations:** ^1^ Department of Pediatrics and Department of Neurology University of Texas Southwestern Medical Center Dallas Texas USA; ^2^ Division of Neurology Children's Hospital of Philadelphia Philadelphia Pennsylvania USA; ^3^ The Epilepsy NeuroGenetics Initiative (ENGIN) Children's Hospital of Philadelphia Philadelphia Pennsylvania USA; ^4^ Department of Biomedical and Health Informatics (DBHi) Children's Hospital of Philadelphia Philadelphia Pennsylvania USA; ^5^ TBC1D24 Foundation Milford Pennsylvania USA; ^6^ Yale Medicine New Haven Connecticut USA; ^7^ Department of Neurology University of Pennsylvania Perelman School of Medicine Philadelphia Pennsylvania USA

**Keywords:** developmental and epileptic encephalopathy, epilepsy, genetics, Human Phenotype Ontology, *TBC1D24*

## Abstract

**Objective:**

Biallelic variants in *TBC1D24* represent a rare cause of epilepsy and neurodevelopmental disorders, including severe developmental and epileptic encephalopathies. Here, we present the first attempt to delineate the longitudinal disease histories and effectiveness of antiseizure medications (ASMs) in *TBC1D24*‐related disorders.

**Methods:**

We performed an analysis of the electronic medical record data of 15 individuals with *TBC1D24*‐related disorders. Using the Human Phenotype Ontology, we recorded neurological histories and medication responses across 197 patient‐years of information.

**Results:**

Individuals with *TBC1D24*‐related disorders presented with a range of seizure types with a median age at seizure onset of 3 months—most frequently (73%) with focal myoclonic seizures both sparing and impairing consciousness. We report the maximum prevalence (MP) of various features as percentages of individuals reporting a given phenotype at that time point, compared to all those with available data at that time point. MP of focal seizures was at 6.25 and 7.75 years of age (88%), myoclonic seizures (focal and generalized) between 9 and 10 years of age (80%), and status epilepticus at 9 and 11 months of age (90%). Individuals also presented with a range of movement disorders. The MP of non‐epileptic myoclonus was 100% at 1 and 17 months of age, tremor at 14 months of age (67%), ataxia at 7.25 years of age (45%), and episodic hemiplegia at 3.25 years of age (20%). The use of phenobarbital, oxcarbazepine, and topiramate showed the most promise in seizure management when compared to other ASMs. Everolimus, phenobarbital, and oxcarbazepine proved more effective in maintaining seizure freedom or reducing seizure frequencies in focal and myoclonic seizures compared to other ASMs.

**Significance:**

*TBC1D24*‐related disorders are characterized by severe and pharmacoresistant epilepsy, with status epilepticus, focal seizures, and myoclonic seizures early in life. This study offers novel insights into the longitudinal disease course and treatment response in *TBC1D24*‐related disorders, a critical first step toward clinical trial readiness.

**Key points**

- *TBC1D24*‐related disorders encompass a wide spectrum of neurological phenotypes, but the longitudinal trajectories remain unexplored.
- Homozygous p.Pro282Arg variants were found in five individuals of Mexican descent, making it a likely founder mutation.
- Early onset and high frequency of seizures and movement disorders reflect the burden and complexity of the condition over time.
- The refractory and unpredictable nature of *TBC1D24* epilepsy necessitates the development of precision medicine approaches.

## 1 INTRODUCTION

A spectrum of neurodevelopmental disorders has been associated with biallelic variants in the *TBC1D24* gene, including infantile myoclonic epilepsy and developmental and epileptic encephalopathies (DEEs). In addition, *TBC1D24*‐related disorders are known to include non‐syndromic hearing loss and DOORS (deafness, onychodystrophy, osteodystrophy, retardation (intellectual disability), and seizures) syndrome, a complex syndromal condition characterized by deafness, onychodystrophy, osteodystrophy, intellectual disabilities, and seizures.^1^, ^2^, ^3^, ^4^ Additional clinical presentations have emerged over the last decade, including focal myoclonic seizures with migrating involvement of extremities^5^ and a spectrum of movement disorders.^6^ However, the evolution over time of specific clinical features in *TBC1D24*‐related disorders remains largely unknown.

Despite the limited knowledge of longitudinal phenotypes in *TBC1D24* epilepsy, the role of the *TBC1D24* gene has been studied extensively at the cellular level. The gene encodes the N‐terminal Tre2‐Bub2‐Cdc16 (TBC) domain involved in the regulation of vesicle trafficking. In vivo demonstration of *TBC1D24* haploinsufficiency in mouse models led to enlarged neuronal endosomes, impaired endocytic functioning, and reduced spontaneous neurotransmission.^7^ Prior studies on underlying disease mechanisms yielded evidence of oxidative stress intolerance leading to dysregulation and impairment of synaptic vesicle trafficking in developing neurons.^8^ Although the molecular underpinnings and initial genotype–phenotype associations of *TBC1D24*‐related disorders have been described, individual phenotypic trajectories of these conditions remain unexplored.

Here, we leverage electronic medical record (EMR) data to characterize the longitudinal clinical features and treatment response in 15 individuals with *TBC1D24*‐related epilepsy. We find that *TBC1D24*‐related disorders present with a range of seizure types with specific age‐related patterns and frequent episodes of status epilepticus. In addition, individuals present with a wide range of neurological features beyond seizures.

## 2 MATERIALS AND METHODS

### 2.1 Defining the cohort

We included 15 individuals with *TBC1D24*‐related disorders who were seen at the University of Texas Southwestern Medical Center (UTSW, *n* = 9) and the Epilepsy NeuroGenetics Initiative at the Children's Hospital of Philadelphia (CHOP, *n* = 6). All individuals were included in all analyses, including two infants with very severe presentations who both died in the first year of life. *TBC1D24* variants were reviewed by a genetic counselor who specialized in neurogenetics and classified using the American College of Medical Genetics and Genomics (ACMG) criteria. Variants of uncertain significance were confirmed to be causative through clinical correlation with distinct *TBC1D24*‐related epilepsy presentations (Table S1). The study was approved by the relevant institutional review boards.

### 2.2 Extracting and mapping phenotypic information

To reconstruct longitudinal clinical trajectories in *TBC1D24*‐related disorders, we retrieved time‐stamped phenotypic information from the EMR. For each individual, the presence or absence (if known) of phenotypes described in the clinical records were recorded in monthly increments using the Human Phenotype Ontology (HPO) to ensure standardization of the collected data, as performed previously.^9^, ^10^ Each symptom recorded in the EMR was assigned a maximally specific HPO term. In classifying any clinical features that may present similarly, for example, myoclonic seizures and myoclonus, we followed the documentation provided in the clinical record. No event was assumed to be a seizure unless it was explicitly called as such. We discuss seizure types in our cohort based on the International League Against Epilepsy 2025 nomenclature.

Following the manual extraction of HPO‐translated terms, we expanded the phenotyping by assigning all higher‐level terms to each base term using the branching logic of the HPO. For example, if an individual had a focal myoclonic seizure (HP:0011166) recorded in the chart, this information led us to infer that they also had a myoclonic seizure (HP:0032794) and a focal seizure (HP:0011153). Through this approach, we were able to assign both granular and general features in individuals with *TBC1D24*‐related disorders. In recording longitudinal frequencies of specific epilepsy and movement disorder phenotypes, we report the proportion of individuals with the specific phenotype compared to those with the broader category (seizure HP:0001250 or abnormal central motor function HP:0011442) at each time point. For example, if at 3 months, eight individuals had seizures, with four of them having focal seizures, the frequency of focal seizures at 3 months was recorded to be 50%.

### 2.3 Reconstructing seizure histories

Seizure histories were reconstructed from the EMRs of all patients in monthly increments. We recorded seizure frequencies (SFs) according to the Epilepsy Learning Health System (ELHS) and Pediatric Epilepsy Learning Health System (PELHS) conventions.^11^ The SF scores included: no seizures (SF score = 0), monthly seizures (SF score = 1), weekly seizures (SF score = 2), daily seizures (SF score = 3), several daily seizures (2–5 per day, SF score = 4), and multiple daily seizures (>5 per day, SF score = 5).

### 2.4 Determining the comparative antiseizure medication (ASM) effectiveness

We extracted the ages at initiation and discontinuation of medications from the EMR to determine when each ASM was prescribed, excluding rescue medications. To determine the comparative ASM effectiveness, we assessed changes in SF scores during the time in which a given medication was prescribed compared to all other time periods, as done by our group previously.^10^, ^12^ We then evaluated the efficacy of each ASM in maintaining seizure freedom or reducing seizure frequencies. ASMs taken by fewer than three patients were excluded from the final analysis.

### 2.5 Statistical analysis

We used the R Statistical Package for all analyses in this study. ASM comparative effectiveness was evaluated using Fisher's exact test with a significance threshold of *p* < .05. For each association, odds ratios (ORs) and 95% confidence intervals (CIs) are provided.

## 3 RESULTS

### 3.1 *TBC1D24* ‐related disorders span a wide genetic and phenotypic spectrum

We analyzed the longitudinal clinical data of 15 individuals with *TBC1D24*‐related disorders across 197 patient years and 1797 HPO terms (mean = 119.8, interquartile range [IQR] = 92.5–153.5), (Figure S1). The most common clinical traits present in our cohort included focal seizures (100%), movement disorder (100%), focal or generalized myoclonic seizures (100%), status epilepticus (93%), neurodevelopmental abnormality (93%), bilateral tonic–clonic seizures (87%), myoclonus (80%), generalized seizures (80%), and tremor (67%). Notable features observed less frequently included ataxia (47%), dystonia (47%), episodic hemiplegia (40%), hearing impairment (40%), abnormalities of eye movement (40%), sleep abnormality (33%), episodic ataxia (33%), and paroxysmal dystonia (20%).

**FIGURE 1:**
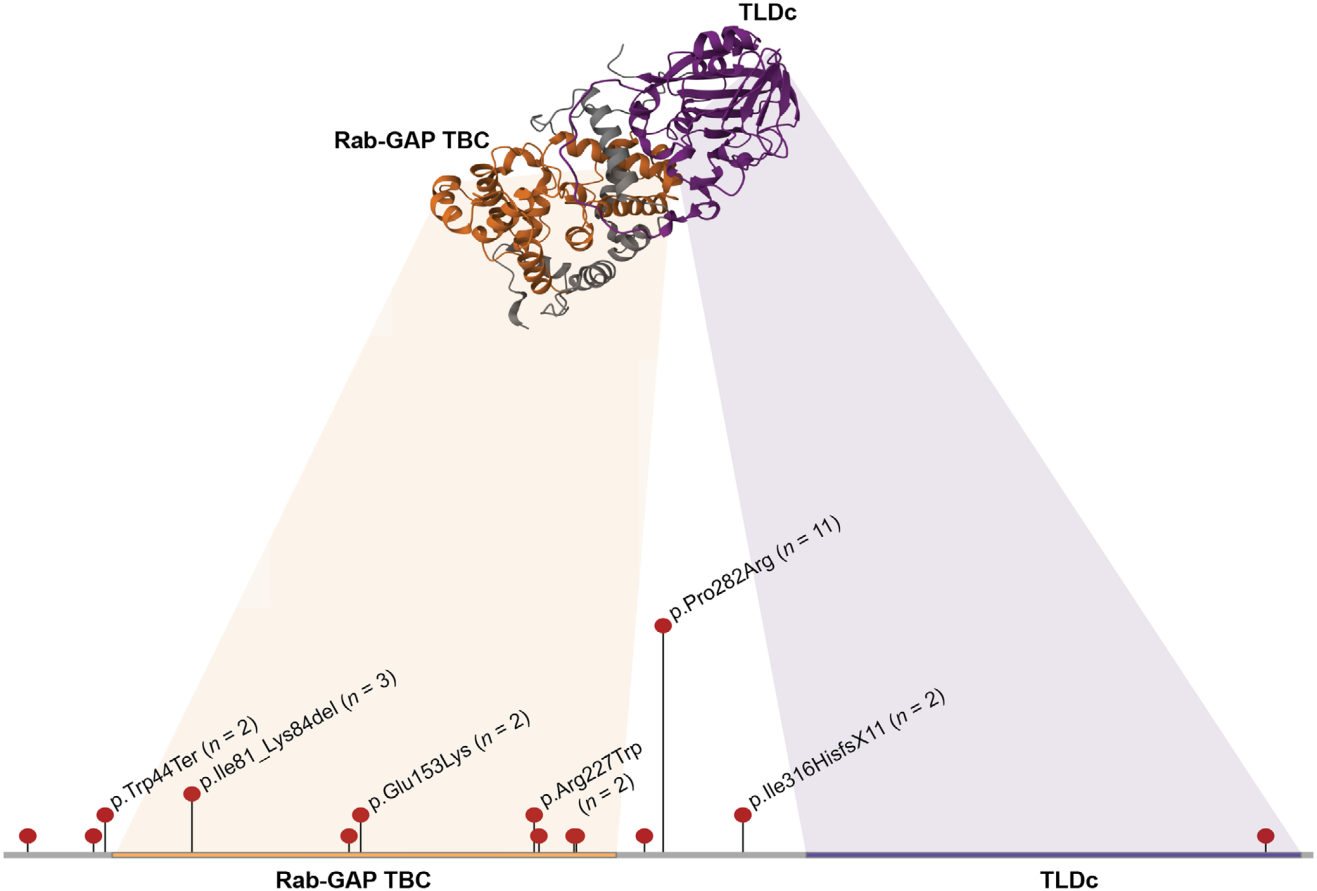
Distribution of *TBC1D24* variants. Each N corresponds to the count of a given variant in our cohort. The AlphaFold representation of the TBC1D24 protein displays its two functional domains.

We identified the following variants in *TBC1D24* in several individuals: p.Pro282Arg (*n* = 11), p.Ile81_Lys84del (*n* = 3), p.Trp44Ter (*n* = 2), p.Glu153Lys (*n* = 2), p.Arg227Trp (*n* = 2), and p.Ile316HisfsX11 (*n* = 2), where each *n* corresponds to the count of a given variant in our cohort (Table 1; Figure 1). When comparing the phenotypic differences between the carriers of two p.Pro282Arg variants to the rest of the cohort, we discovered that these individuals were more likely to present with the following features: dystonia (*p* = .007, OR Inf, 95% CI 1.69‐Inf), growth abnormality (*p* = .017, OR 23.62, 95% CI 1.18–11918.98), and amblyopia (*p* = .022, OR 10.66, 95% CI 1.07‐Inf), (Figure S2).

**TABLE 1:** Cohort overview.

Patient ID	Sex	Variant 1; Variant 2	Latest age at evaluation (months) *age at death	Seizure onset (months)	Broad seizure types
1	M	p.Ile81_Lys84del; p.Cys539Tyr	21 years, 6 months	4	Focal impaired consciousness tonic; generalized tonic; focal tonic; myoclonic; focal impaired consciousness with autonomic features; generalized atonic; bilateral tonic–clonic
2	M	p.Trp44Ter; p.Ile81_Lys84del	19 years, 10 months	4	Generalized tonic; focal impaired consciousness with behavioral arrest; focal impaired consciousness myoclonic; generalized atonic; bilateral tonic–clonic
3	M	p.Ile81_Lys84del; p.Arg274del	3 years, 10 months	4	Focal impaired consciousness myoclonic; bilateral tonic–clonic; focal myoclonic
4	F	p.Trp44Ter; p.Glu153Lys	12 years	2	Focal impaired consciousness myoclonic seizure; focal seizure with automatisms; bilateral tonic–clonic
5	F	p.Pro282Arg; p.Pro282Arg	8 years, 5 months	2	Focal myoclonic; bilateral tonic–clonic
6	F	p.Pro282Arg; p.Pro282Arg	21 years, 3 months	5	Focal impaired consciousness myoclonic; generalized atonic; bilateral tonic–clonic
7	F	p.Pro282Arg; p.Ala244Val	21 years, 6 months	2	Focal impaired consciousness myoclonic; bilateral tonic–clonic focal seizure with automatism
8	M	p.Pro282Arg; p.Pro282Arg	14 years, 8 months	2	Generalized tonic; focal impaired consciousness with behavior arrest; epileptic spasms; focal impaired consciousness myoclonic; bilateral tonic–clonic
9	M	p.Pro282Arg; p.Pro282Arg	4 years, 9 months	4	Generalized myoclonic; bilateral tonic–clonic; generalized atonic; focal impaired consciousness myoclonic
10	F	p.Glu148Ter; p.Glu153Lys	6 years, 3 months	3	Focal impaired consciousness seizure with observable motor phenomena; generalized seizures focal seizures without observable characteristics; myoclonic seizures
11	M	p.Ala39Val; p.Phe229Ser	7 months*	3	Focal seizure with unilateral clonus; myoclonic seizure; focal seizure with observable motor phenomena; bilateral tonic–clonic
12	F	p.I316HfsX11; p.Arg227Trp	21 years, 6 months	2	Focal seizure with observable motor phenomena; generalized seizures; bilateral tonic–clonic; focal impaired consciousness seizure
13	F	p.I316HfsX11; p.Arg227Trp	21 years, 6 months	2	Focal myoclonic seizure with migrating limb involvement; generalized seizure; bilateral tonic–clonic
14	F	p.Pro282Arg; p.Pro282Arg	17 years, 5 months	3	Epileptic spasms; focal seizures; generalized seizures; unknown if focal or generalized myoclonic seizures; atypical absence
15	F	p.Asp11Gly; p.Leu245Pro	7 months*	2	Focal impaired consciousness; unknown if focal or generalized myoclonic; bilateral tonic–clonic; focal clonic seizures; focal myoclonic seizure with migrating limb involvement; epileptic spasms

### 3.2 *TBC1D24* ‐related disorders show distinct temporal phenotypic signatures

**FIGURE 2:**
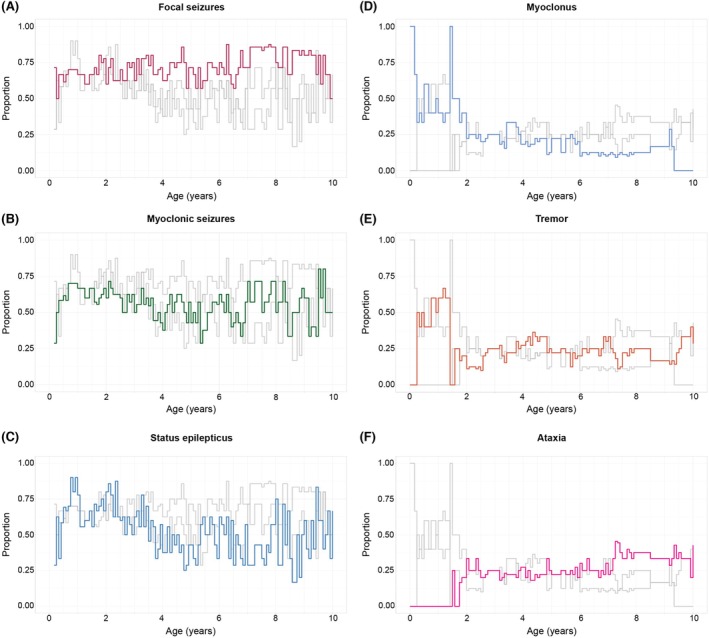
Longitudinal landscape of seizure and movement disorder phenotypes in *TBC1D24*‐related disorders. Individuals with *TBC1D24*‐related disorders present with early‐life epilepsy and movement disorders. Each line corresponds to the proportion of individuals with a given seizure or movement phenotype in each time bin, where colored lines highlight the specified phenotype and the gray lines correspond to the other epilepsy (A–C) and movement (D–F) phenotypes.

By assessing longitudinal clinical data in our cohort, we were able to investigate the progression of key clinical features over time. At all timepoints assessed, a high proportion of individuals with *TBC1D24*‐related disorders had seizures with a high frequency of status epilepticus (Figure 2). In addition, many individuals had neurodevelopmental delay and various movement disorders. Seizures persisted in more than half of the cohort across almost all ages, with the highest proportion observed in the first 3 years of life. Status epilepticus occurred throughout the lifespan, particularly in the first decade of life.

We found unique temporal progression patterns for defined seizure and movement phenotypes of *TBC1D24*‐related disorders (Figure 2). The following maximum symptom prevalences (MPs) are reported as percentages of the cohort actively reporting any movement disorders at a given time point. Movement disorders presented early in infancy with myoclonus (MP 100% at birth, 1, and 17 months) and tremor (MP 67% at 14 months). Other neurological symptoms emerged later in childhood, including ataxia (MP 100% at 19 years) and hemiplegia (MP 25% at 17 years).

**FIGURE 3:**
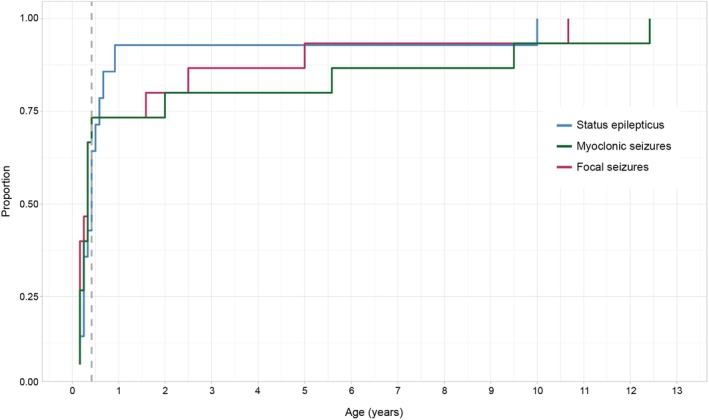
Cumulative seizure onset in *TBC1D24*‐related disorders. All individuals had seizures by 5 months of life (dashed line). Different colors represent different seizure types most commonly occurring in our cohort (focal seizures – maroon, myoclonic seizures – green, status epilepticus – blue).

The median seizure onset for our cohort was 3 months of age, and all individuals had seizures by 5 months of age (Figure 3). Focal‐onset seizures were typically the first seizure type to be reported, and almost 75% of the cohort experienced focal seizures, myoclonic seizures, or status epilepticus by 6 months of age. Initial seizure semiologies at onset included focal myoclonic seizures (with both preserved and impaired consciousness). Overall, focal seizures (particularly focal myoclonic) and status epilepticus predominated throughout the clinical history (Figure 2). Generalized seizures were less frequent in infancy but modestly increased in frequency through toddlerhood (MP 50% at 2 years).

Besides focal myoclonic and generalized tonic–clonic seizures, the cohort's semiologies further diversified through childhood (all listed in Table 1, with detailed semiologies and electroencephalography [EEG] features in Table S2). Other generalized semiologies included: generalized tonic seizures (*n* = 6), generalized atonic seizures (*n* = 5), epileptic spasms (*n* = 4), and atypical absence seizures (*n* = 1). Focal seizure semiologies included: focal impaired consciousness seizures with behavioral arrest (*n* = 4), focal impaired consciousness tonic seizures (*n* = 3), focal seizures with automatisms (*n* = 3), focal seizures with hypoventilation (*n* = 2), focal seizures with auditory phenomena (*n* = 1), and focal seizures with visual phenomena (*n* = 1).

**FIGURE 4:**
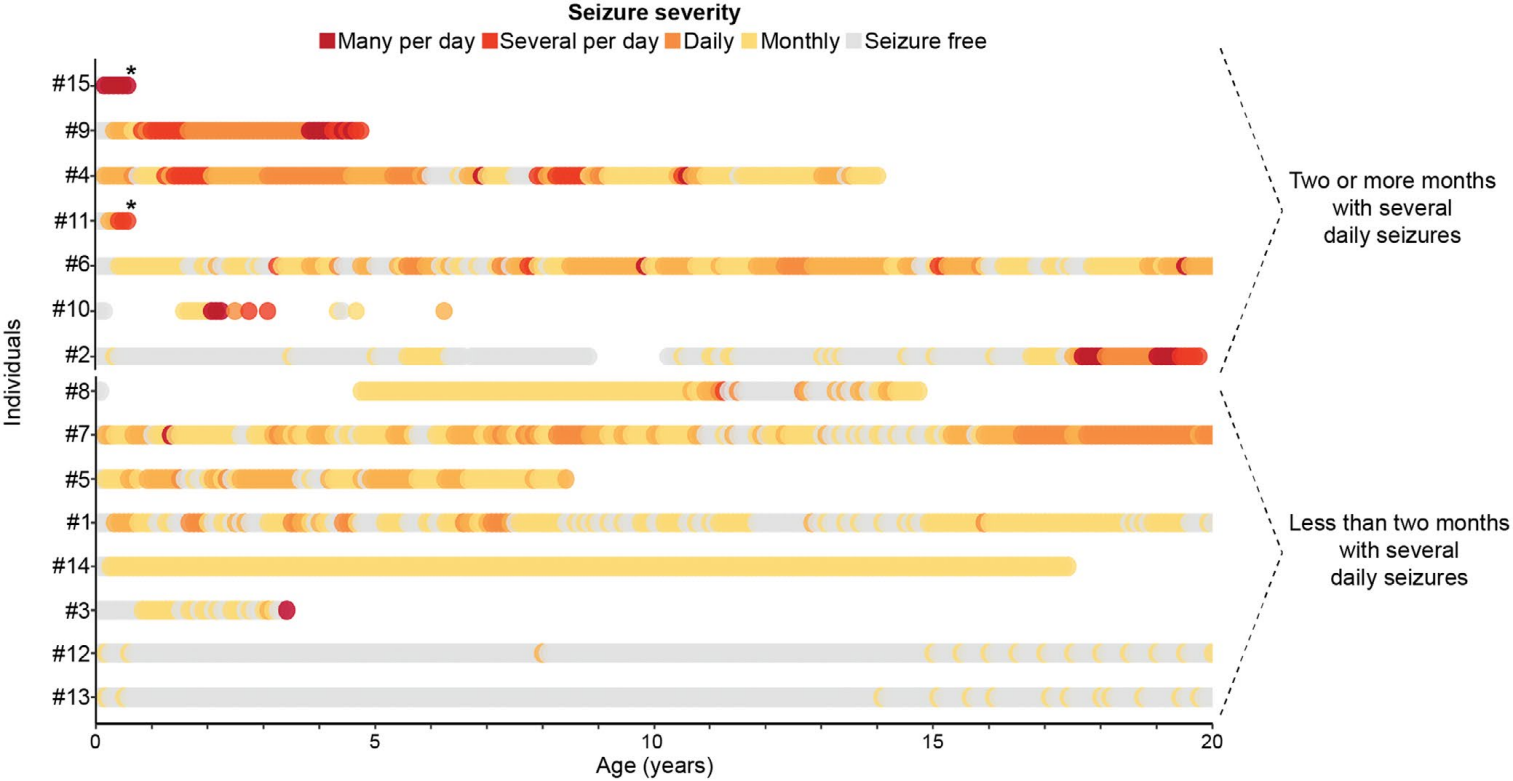
Seizure frequencies in *TBC1D24*‐related disorders. Monthly seizure frequencies reflect differences in seizure trajectories in *TBC1D24* epilepsy. The stars denote the age at death.

Reconstructing seizure histories on a month‐by‐month basis delineated seizure progression in *TBC1D24*‐related epilepsies (Figure 4). The overall highest seizure frequencies and most dynamic changes in seizure frequencies were observed in the first 5 years of life in our cohort. However, during this same age range, we observed prominent differences in seizure severity among the individuals we assessed. We therefore used cumulative seizure frequences to divide the cohort into two equally sized subgroups—individuals with fewer than 2 months of having several (SF score > = 4) daily seizures (*n* = 8) and individuals with at least 2 months of having several daily seizures (*n* = 7). Seizure frequencies varied widely over time in both subgroups, highlighting the complex and unpredictable history of *TBC1D24*‐related disorders. Some individuals (1, 2, and 8) experienced the recurrence of frequent seizures after long periods of seizure freedom.

### 3.3 Neurodevelopmental features and other neurological features in *TBC1D24* ‐related disorders

In our cohort of 15 individuals, 93% experienced neurodevelopmental differences. Motor delay and speech delay were documented in 47% and 60%, respectively. Six individuals (40%) exhibited behavioral challenges, including autistic features, aggression, and self‐injurious behaviors. In our cohort, 40% were formally diagnosed with intellectual disability (moderate *n* = 2, mild *n* = 2, unspecified *n* = 2). Only two of our patients (2 and 4) required permanent feeding tube placement. Those same two children were also the only individuals who were documented to remain non‐verbal past toddlerhood. Sensorineural hearing impairment was reported in 40% of our patients. Eye abnormalities resulting in visual impairment were seen in 67%, including myopia (27%), strabismus (27%), alternating exotropia (13%), amblyopia (20%), and legal blindness in one patient.

Other neurodevelopmental differences, such as abnormal communication (67%), speech delay (60%), and language impairment (60%) were also observed in our cohort. A subset of individuals had abnormal muscle tone (53%); other neurological features, such as ataxia (47%) and dystonia (47%), were common in our cohort as well. Three individuals (13%) had hyperkinetic movements.

### 3.4 Early death in *TBC1D24* epilepsy: Seizure burden and respiratory depression

Two unrelated individuals from our cohort died at 7 months of age, adding to prior reports of fatal medically refractory seizures in toddlers and infants with *TBC1D24* epilepsy.^3^, ^4^, ^13^ Individual 11 was admitted due to increased seizure frequency and refractory status epilepticus in the setting of a parainfluenza infection. During admission, the patient required escalation of seizure treatment and intubation for acute respiratory failure. Status epilepticus was refractory to treatment with ketamine infusion, and compassionate care was pursued with opiates, barbiturates, and benzodiazepines. Patient 15 was admitted at 7 months of age due to increased seizure frequency and status epilepticus with focal‐to‐bilateral tonic–clonic seizures. Despite the use of multiple ASMs, status epilepticus could not be controlled and led to intubation due to respiratory compromise. Care was redirected after several unsuccessful attempts at extubation. Both cases demonstrate the refractory nature of status epilepticus in *TBC1D24*‐related disorders in infancy, which led to compassionate care decisions in both scenarios.

### 3.5 ASM treatment outcomes reveal the pharmacoresistance of *TBC1D24* epilepsy

**FIGURE 5:**
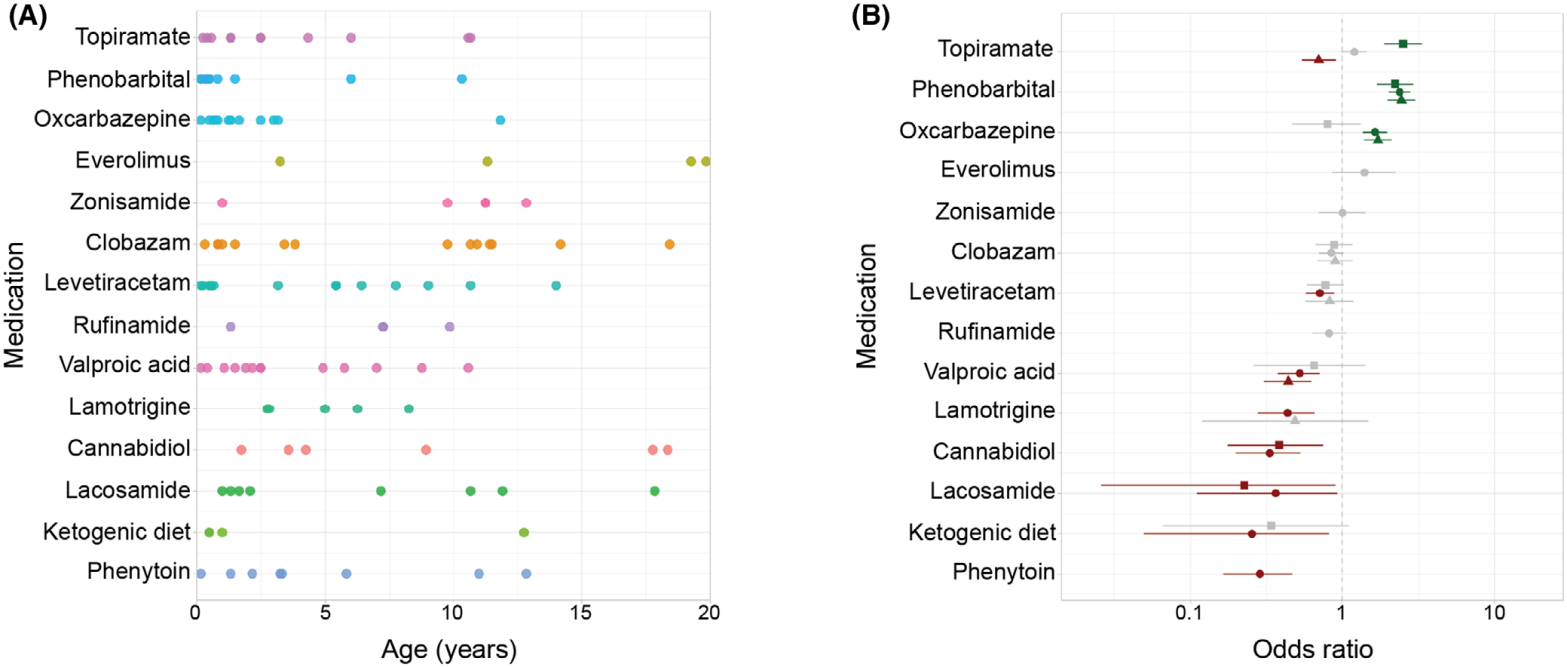
Antiseizure medication (ASM) landscape and ASM effectiveness analysis. Prescription patterns over time (A). The effectiveness of each ASM for individuals with less severe seizure frequencies (<2 months with several daily seizures) is denoted by triangles, for individuals with more severe seizure frequencies (≥2 months with several daily seizures) is denoted by squares, and for the entire cohort is denoted by dots (B).

We reconstructed the prescription patterns of 14 ASMs in our cohort of 15 individuals with *TBC1D24*‐related disorders (Figure 5A). The most frequently used medications included levetiracetam (93%), clobazam (80%), topiramate (73%), oxcarbazepine (67%), valproic acid (67%), and phenobarbital (60%). Multiple ASMs were concurrently prescribed, highlighting the drug‐refractory character of *TBC1D24*‐related epilepsy.

Through a combined analysis of medication prescription data and monthly seizure frequencies, we assessed the effectiveness of ASMs in managing seizures in *TBC1D24*‐related disorders (Figure 5B). We evaluated the ability of different ASMs to reduce seizure frequency or maintain seizure freedom. Phenobarbital (*p* = 2.06 × 10^−25^, OR 2.38, 95% CI 2.02–2.80) and oxcarbazepine (*p* = 1.76 × 10^−7^, OR 1.64, 95% CI 1.36–1.98) were superior to the other ASMs we assessed in this analysis. When the analysis was limited to individuals with more severe seizures (at least 2 months with several daily seizures, Figure 4), topiramate (*p* = 2.17 × 10^−10^, OR 2.51, 95% CI 1.88–3.34) and phenobarbital (*p* = 7.11 × 10^−9^, OR 2.22, 95% CI 1.69–2.92) were superior in reducing seizure frequencies or maintaining seizure freedom compared to all other ASMs, whereas cannabidiol (*p* = .002, OR .38, 95% CI .18–.75) and lacosamide (*p* = .03, OR .23, 95% CI .03–.90) were inferior. Of note, in the subgroup of individuals with fewer seizures (<2 months with several daily seizures, Figure 4), topiramate (*p* = .006, OR .70, 95% CI .54–.91) was less effective than other ASMs, as was valproic acid (*p* = 6.05 × 10^−7^, OR .44, 95% CI .30–.62). In contrast, phenobarbital (*p* = 1.55 × 10^−17^, OR 2.45, 95% CI 1.99–3.02) and oxcarbazepine (*p* = 3.32 × 10^−7^, OR 1.72, 95% CI 1.39–2.12) were more effective in seizure management compared to other ASMs in this subgroup of individuals with less frequent seizures. These disparate results demonstrate a complex pattern of medication response in *TBC1D24*‐related disorders and may serve as an initial framework to generate evidence for optimized seizure management.

In addition to seizure severity subgroups, we analyzed the ASM response based on seizure type (Figure S3). Phenobarbital (*p* = 1.50 × 10^−9^, OR 1.70, 95% CI 1.43–2.02), oxcarbazepine (*p* = 2.34 × 10^−9^, OR 1.69, 95% CI 1.42–2.01), topiramate (*p* = .010, OR 1.28, 95% CI 1.06–1.54), and everolimus (*p* = .011, OR 1.69, 95% CI 1.12–2.56) were superior in managing focal seizures compared to other ASMs. Myoclonic seizures were more effectively treated with phenobarbital (*p* = 1.62 × 10^−15^, OR 2.10, 95% CI 1.75–2.52), oxcarbazepine (*p* = 2.91 × 10^−11^, OR 1.95, 95% CI 1.60–2.38), and everolimus (*p* = 5.33 × 10^−4^, OR 2.02, 95% CI 1.34–3.04) compared to other ASMs. For other seizure types, medication prescriptions were too rare or documented seizure frequencies too sparse for this analysis to be performed. These results demonstrate that similar ASMs may be useful in controlling a range of seizure types in *TBC1D24*‐related disorders.

## 4 DISCUSSION

Through a standardized approach to map and reconstruct natural disease history, we present a first effort to delineate the longitudinal clinical histories of 15 individuals with *TBC1D24*‐related disorders, illuminating previously unexplored aspects of this condition. We found a high frequency of focal myoclonic seizures, status epilepticus, and movement disorders in early life, highlighting the overall disease burden of *TBC1D24*‐related disorders. In addition, we identified ASMs that showed the most promise in managing *TBC1D24* epilepsy.

The most commonly occurring variant, p.Pro282Arg, has been described previously in a heterozygous state in two individuals with seizures starting in early infancy.^1^, ^14^ A total of six individuals in our cohort were homozygous for the p.Pro282Arg variant. This pathogenic variant is likely associated with a founder effect, as all the affected patients were of Mexican descent. Corroborating the two prior case reports,^1^, ^14^ all six affected patients had an onset of refractory epilepsy and focal status epilepticus in infancy. Each of these six individuals subsequently had significant developmental delay, seizure semiologies spanning the known spectrum of *TBC1D24*‐related epilepsies, exertion‐associated movement disorders, and varying degrees of visual impairment. However, in contrast to other *TBC1D24* variants, we found relative sparing of hearing on audiologic assessments, with only two exceptions. Individual 8 recurrently experienced transient hearing loss after his seizures in adolescence. In addition, Individual 14 had unspecified hearing impairment.

The median onset of epilepsy in our cohort was 3 months of age, which is consistent with prior reports.^1^, ^2^, ^3^, ^4^ In a significant subset of individuals, seizure frequencies increased dramatically after onset. In a murine model of *TBC1D24*‐related epilepsy, subjects developed typically through the second week of life but experienced unprovoked seizures leading to death in the third week of life.^15^ This may suggest that *TBC1D24* gene expression is low during fetal development and the early postnatal period, or that *TBC1D24* deficiency can be compensated until mid‐infancy. Such a temporal pattern of seizures prompts consideration of an underlying biological basis in *TBC1D24* epilepsy that could potentially be exploited therapeutically—perhaps with gene replacement—if the disorder can be identified sufficiently early.

Mortality in *TBC1D24*‐related disorders appears to be most prominent in infancy and early childhood, as demonstrated by both individuals in our cohort who died at 7 months and the nine individuals reported in the literature to have died in early childhood.^3^, ^6^, ^13^, ^14^ However, there does not appear to be an increased rate of premature mortality beyond infancy and early childhood in *TBC1D24*‐related disorders, with the exception of a 21‐year‐old individual who died of uncertain causes.^6^ This finding suggests that infancy and early childhood are particularly vulnerable periods for individuals with *TBC1D24*‐related disorders—a critical consideration for counseling families and medical management. Furthermore, this vulnerable time may also represent the major treatment window during which care for individuals with *TBC1D24*‐related disorders can be improved.

Seizures were difficult to control in most individuals in our cohort, highlighting the refractory nature of *TBC1D24*‐related epilepsy. Various reports in the literature have suggested the relative benefit of several ASMs, including phenobarbital, phenytoin, levetiracetam, valproate, and clonazepam in *TBC1D24*‐related seizures.^6^ Phenytoin, levetiracetam, and valproate, have demonstrated less efficacy in our cohort. Our results corroborate prior reports of relative response to phenobarbital and suggest potential clinical utility of topiramate, oxcarbazepine, and everolimus. These findings add to the complexities of interpreting medication response in a rare condition with a highly variable natural history, indicating that standardization of documenting disease trajectories may be beneficial. We performed a systematic analysis based on reconstructed seizure and medication histories as conducted previously for *STXBP1*‐related disorders,^10^, ^12^, ^16^ *SYNGAP1*‐related disorders,^17^ and *SCN8A*‐related disorders.^18^ Our method relies on provider reports of seizure frequencies that were assessed retrospectively from medical records, a technique that allows for secondary use of clinical data at various levels of granularity, including missing data.^10^, ^16^ Of note, we did not exclude the two infants with severe epilepsy and early death from these analyses, which should be considered when translating our findings into clinical practice. Although the relative superiority and inferiority of various ASMs in our study may be due to the fluctuating baseline in *TBC1D24*‐related disorders, this analysis represents a framework that can be expanded with additional data, allowing for comparison to other conditions that have been reconstructed using similar methods.

Several patients in our cohort exhibited focal myoclonic seizures without EEG correlate, an unusual clinical feature reported previously in *TBC1D24*‐related disorders.^13^ We observed that these events typically aborted in response to the administration of rescue benzodiazepines. These individuals, like all our other patients, also intermittently experienced non‐epileptic myoclonus. Acknowledging the difficulty in differentiating these two entities, we assigned the symptoms as they were documented in the electronic medical record per the clinical judgment of the treating neurologist and concurrent EEG reports when available. The co‐occurrence of epileptic and non‐epileptic myoclonus in *TBC1D24*‐related disorders is perplexing, and both forms of myoclonus may appear independently. Confirmed non‐epileptic myoclonus persisting into school age was reported previously in siblings with *TBC1D24*‐related disorders.^19^ In addition, a separate individual with a *TBC1D24*‐related disorder was found to have non‐epileptic migratory myoclonus emerging in infancy.^20^ Both forms of myoclonus coexist in *TBC1D24*‐related disorders, but distinguishing them clinically in an individual patient can prove challenging, especially when relying on medical records alone. Although resolving this clinical dilemma is beyond the scope of this study, we acknowledge the diagnostic complexity of *TBC1D24*‐related myoclonus, as failure to do so can impede effective treatment. Further work to elucidate this distinction would also refine our understanding of other genetic epilepsies that have been associated with non‐epileptic myoclonus.^21^, ^22^

In addition to the prominent seizure and movement phenotypes, we observed that many individuals in our cohort had visual and hearing impairment. The high rate of sensorineural hearing impairment (*n* = 6, 40%) has been reported previously in *TBC1D24*‐related disorders.^23^ However, the high rate of visual impairment (*n* = 10, 67%) in our cohort is novel and may represent a previously overlooked feature of these conditions, or a specific feature of homozygous carriers of the p.Pro282Arg variant. Given that many developmental attainments are highly dependent on visual input, further delineating visual symptoms in *TBC1D24‐*related disorders will be critical to outline the complex neurological features of this condition.

Certain considerations are necessary when interpreting our findings. Although EMR data from two tertiary referral centers allow for detailed phenotypic reconstruction that would not be otherwise attainable, our centers' high specialization level introduces a potential selection bias for more severe phenotypes. Our retrospective chart review method necessitates reliance on what was discussed and documented during the clinical encounter without enabling standardized reporting of certain outcome measures. Furthermore, the high burden of epilepsy dominated the focus of their clinical encounters with neurology and other specialties, likely precluding detailed and standardized documentation of less acute aspects of their condition, namely the specific timing and nature of developmental delays. Thus, detailed information regarding neurodevelopmental outcomes and the progression of seizure types over time was sometimes difficult to discern from the clinical records. Despite these limitations, we have chosen to report developmental delays with that lower degree of specificity to inform the overall longitudinal clinical picture and contribute to prognostic discussions. Although all available data were included in the analyses, we acknowledge that retrospective real‐world datasets often contain missing information. Finally, one‐third of our cohort carried homozygous p.Pro282Arg variants. Although this limits the heterogeneity of individuals in our study, it also provides valuable insights into the phenotypic spectrum of this specific variant.

## 5 CONCLUSIONS

In summary, we describe the longitudinal clinical histories of 15 individuals with *TBC1D24*‐related epilepsy. After the explosive onset of seizures in early infancy, many individuals exhibit prominent neurodevelopmental delays, drug‐refractory epilepsy, and various movement disorders, including non‐epileptic myoclonus. By providing a standardized framework to catalog neurological features and treatments, we are taking the first step toward deciphering the complex natural history of *TBC1D24‐*related disorders, one of the most enigmatic genetic epilepsies characterized to date. The results from our study will inform the development of meaningful outcome measures that can be assessed prospectively to advance clinical trial readiness in *TBC1D24‐*related disorders.

## AUTHOR CONTRIBUTIONS

Conceptualization: J.L.M., I.H., S.M.R., D.A., D.S., S.A., E.M., and J.H.M. Data Curation: E.M., B.B., J.N., J.H.M., J.L.M., D.A., and D.S. Formal Analysis: J.H.M. Funding Acquisition: J.L.M. and I.H. Supervision: J.L.M., I.H., and D.A. Writing—Original Draft Preparation: E.M., J.H.M., and B.B. Writing—Review & Editing: E.M., J.H.M., B.B., J.N., S.M.R., D.S., D.A. I.H., and J.L.M.

## FUNDING INFORMATION

J.L.M. was supported by a University of Pennsylvania Orphan Disease Center Grant, in partnership with the *TBC1D24* Foundation, and the National Institute of Neurological Disorders and Stroke (K23 NS140491‐01A1). I.H. was supported by the National Institute of Neurological Disorders and Stroke (R01 NS127830‐01A1, R01 NS131512‐01, and K02 NS112600). This work was also supported by intramural funds of the Children's Hospital of Philadelphia through the Epilepsy NeuroGenetics Initiative (ENGIN).

## CONFLICT OF INTEREST STATEMENT

The authors declare no competing interests.

## ETHICS STATEMENT

We confirm that we have read the Journal's position on issues involved in ethical publication and affirm that this report is consistent with those guidelines.

## Supporting information


Table S1.

## Data Availability

De‐identified primary data are available upon request from the corresponding author.
