# Supplementary material for: Clinical trajectories and medication response in TBC1D24‐related epilepsies
Source: Epilepsia. 2025 Nov 10;67(3):1386–97. doi: 10.1111/epi.70013 (PMC13007820; doi:10.1111/epi.70013)

**Supplementary Table 1. Variant classification.**

| ***TBC1D24* variant** | **Variant classification** |
| --- | --- |
| p.Ile81_Lys84del | Pathogenic |
| p.Cys539Tyr | Uncertain Significance |
| p.Trp44Ter | Pathogenic |
| p.Arg274del | Uncertain significance |
| p.Glu153Lys | Likely pathogenic |
| p.Pro282Arg | Pathogenic |
| p.Ala244Val | Pathogenic |
| p.Glu148Ter | Pathogenic |
| p.Ala39Val | Pathogenic |
| p.Phe229Ser | Pathogenic |
| p.Ile316HisfsX11 | Pathogenic |
| p.Arg227Trp | Pathogenic |
| p.Asp11Gly | Pathogenic |
| p.Leu245Pro | Uncertain significance |

**Supplementary Table 2. Seizure phenotypes and EEG features of the cohort.**

| **ID** | **Variant 1; Variant 2** | **Seizure type(s) at the month of seizure onset** | **EEG features** | **Mean seizure frequency score^1^** |
| --- | --- | --- | --- | --- |
| 1 | p.Ile81_Lys84del; p.Cys539Tyr | Focal preserved consciousness myoclonic seizure | EEG with abnormally slow frequencies  EEG with generalized slow activity  EEG with continuous slow activity  EEG with intermittent slow activity  Interictal EEG abnormality  EEG with focal epileptiform discharges  EEG with generalized epileptiform discharges  EEG with spike-wave complexes  EEG with polyspike wave complexes  Absent posterior alpha rhythm | 1.29 |
| 2 | p.Trp44Ter; p.Ile81_Lys84del | Unclassified | EEG with abnormally slow frequencies  EEG with generalized slow activity  EEG with continuous slow activity  EEG with intermittent slow activity  Interictal EEG abnormality  EEG with focal epileptiform discharges  EEG with generalized epileptiform discharges  EEG with spike-wave complexes  EEG with polyspike wave complexes  Absent posterior alpha rhythm | 2.36 |
| 3 | p.Ile81_Lys84del; p.Arg274del | Focal preserved consciousness myoclonic seizure | Interictal EEG abnormality  EEG with abnormally slow frequencies  EEG with generalized slow activity  EEG with continuous slow activity  EEG with focal epileptiform discharges  Absent posterior alpha rhythm | 1.87 |
| 4 | p.Trp44Ter; p.Glu153Lys | Focal impaired consciousness myoclonic seizure, focal impaired consciousness seizure with hypoventilation | Interictal EEG abnormality  EEG with abnormally slow frequencies  EEG with generalized slow activity  EEG with intermittent slow activity  EEG with continuous slow activity  EEG with generalized epileptiform discharges  EEG with spike-wave complexes  EEG with focal epileptiform discharges  Absent posterior alpha rhythm | 2.09 |
| 5 | p.Pro282Arg; p.Pro282Arg | Focal myoclonic seizure | EEG with abnormally slow frequencies  EEG with generalized slow activity  EEG with intermittent slow activity  EEG with continuous slow activity  Interictal EEG abnormality  EEG with focal epileptiform discharges  EEG with generalized epileptiform discharges  EEG with spike-wave complexes | 1.53 |
| 6 | p.Pro282Arg; p.Pro282Arg | Focal preserved consciousness myoclonic seizure | EEG with abnormally slow frequencies  EEG with generalized slow activity  EEG with intermittent slow activity  EEG with continuous slow activity  Interictal EEG abnormality  EEG with generalized epileptiform discharges  EEG with spike-wave complexes  EEG with focal epileptiform discharges | 1.72 |
| 7 | p.Pro282Arg; p.Ala244Val | Focal preserved consciousness myoclonic seizure, focal impaired consciousness myoclonic seizure | Interictal EEG abnormality  EEG with abnormally slow frequencies  EEG with generalized slow activity  EEG with intermittent slow activity  EEG with continuous slow activity  EEG with generalized epileptiform discharges  EEG with spike-wave complexes  EEG with polyspike wave complexes  EEG with focal epileptiform discharges  Absent posterior alpha rhythm | 1.86 |
| 8 | p.Pro282Arg; p.Pro282Arg | Generalized tonic seizure, epileptic spasms | EEG with abnormally slow frequencies  EEG with generalized slow activity  EEG with continuous slow activity  Interictal EEG abnormality  Absent posterior alpha rhythm  EEG with focal epileptiform discharges  EEG with generalized epileptiform discharges  EEG with spike-wave complexes  EEG with polyspike wave complexes | 1.21 |
| 9 | p.Pro282Arg; p.Pro282Arg | Bilateral tonic-clonic seizure, focal impaired consciousness myoclonic seizure, focal preserved consciousness myoclonic seizure | EEG with abnormally slow frequencies  EEG with generalized slow activity  EEG with continuous slow activity | 3.37 |
| 10 | p.Glu148Ter; p.Glu153Lys | Focal preserved consciousness myoclonic seizure | EEG with abnormally slow frequencies  Interictal EEG abnormality  EEG with focal epileptiform discharges | 2.40 |
| 11 | p.Ala39Val; p.Phe229Ser | Focal seizure with bilateral motor phenomena | Interictal EEG abnormality | 3.20 |
| 12 | p.Ile316HisfsX11; p.Arg227Trp | Myoclonic seizure, focal impaired consciousness seizure with observable manifestations | Interictal EEG abnormality  EEG with generalized epileptiform discharges  EEG with spike-wave complexes | 1.06 |
| 13 | p.Ile316HisfsX11; p.Arg227Trp | Focal seizure with myoclonus and migratory limb involvement, bilateral tonic-clonic seizure | EEG data unavailable | 1.00 |
| 14 | p.Pro282Arg; p.Pro282Arg | Focal preserved consciousness myoclonic seizure | EEG data unavailable | 1.00 |
| 15 | p.Asp11Gly; p.Leu245Pro | Focal impaired consciousness seizure with unresponsiveness, focal seizure with motor phenomena | Interictal EEG abnormality  EEG with focal epileptiform discharges  EEG with focal sharp waves  EEG with central epileptiform discharges  EEG with central sharp waves  EEG with temporal epileptiform discharges  EEG with temporal sharp waves  EEG with parietal epileptiform discharges  EEG with parietal sharp waves  EEG with abnormally slow frequencies  EEG with focal slow activity  EEG with generalized epileptiform discharges | 5.00 |

^1^ Mean seizure frequency was obtained by averaging all non-zero seizure frequencies across each individual’s lifespan.

**Supplementary Figure 1. HPO terms per individual.**


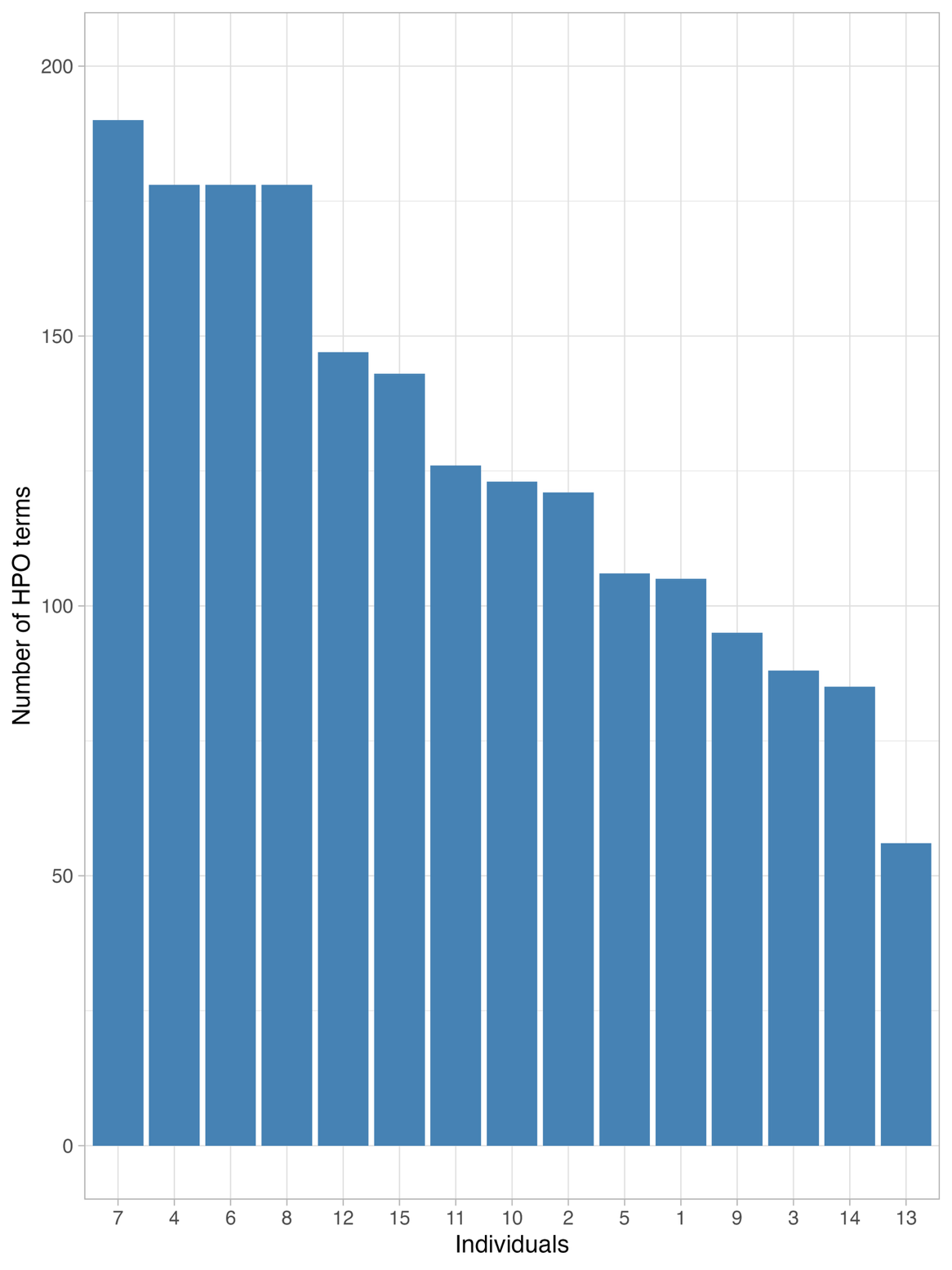


**Supplementary Figure 2. The phenotypic comparison of carriers of two p.Pro282Arg variant carriers (n = 5) to the rest of the cohort.**


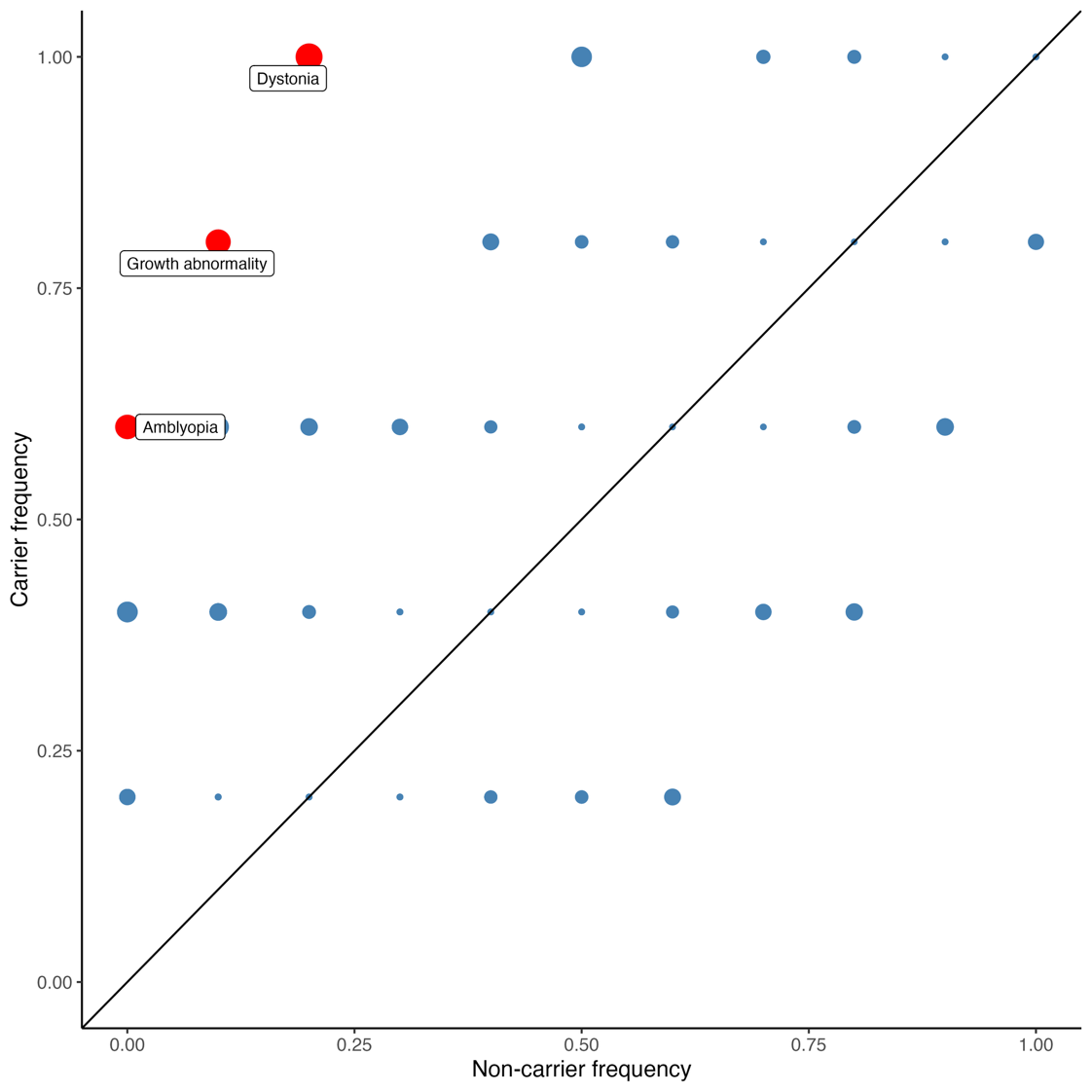


**Supplementary Figure 3. ASM comparative effectiveness for specific seizure subtypes.**


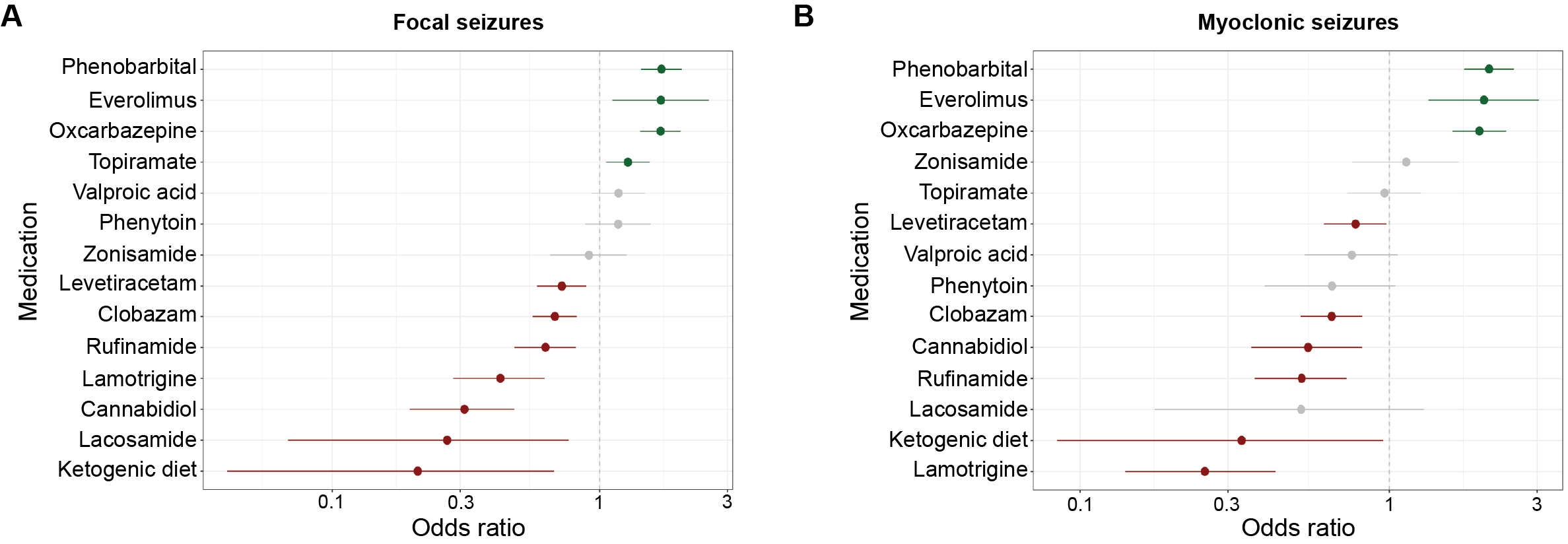

Supplement: Supplementary file 1 — Table S1. [file EPI-67-1386-s001.docx]
